# Exploring approaches to patient safety: the case of spinal manipulation therapy

**DOI:** 10.1186/s12906-016-1149-2

**Published:** 2016-06-02

**Authors:** Linda Rozmovits, Silvano Mior, Heather Boon

**Affiliations:** Qualitative research consultant, Toronto, ON Canada; Canadian Memorial Chiropractic College, Toronto, ON Canada; Leslie Dan Faculty of Pharmacy, University of Toronto, 144 College Street, Toronto, ON M5S 3M2 Canada

**Keywords:** Safety culture, Spinal manipulation, Chiropractic, Physiotherapy

## Abstract

**Background:**

The purpose of this study was to gain insight into the current safety culture around the use of spinal manipulation therapy (SMT) by regulated health professionals in Canada and to explore perceptions of readiness for implementing formal mechanisms for tracking associated adverse events.

**Methods:**

Fifty-six semi-structured telephone interviews were conducted with professional leaders and frontline practitioners in chiropractic, physiotherapy, naturopathy and medicine, all professions regulated to perform SMT in the provinces of Alberta and Ontario Canada. Interviews were digitally audio-recorded for verbatim transcription. Transcripts were entered into HyperResearch software for qualitative data analysis and were coded for both anticipated and emergent themes using the constant comparative method. A thematic, descriptive analysis was produced.

**Results:**

The safety culture around SMT is characterized by substantial disagreement about its actual rather than putative risks. Competing intra- and inter-professional narratives further cloud the safety picture. Participan*t*s felt that safety talk is sometimes conflated with competition for business in the context of fee-for-service healthcare delivery by several professions with overlapping scopes of practice. Both professional leaders and frontline practitioners perceived multiple barriers to the implementation of an incident reporting system for SMT.

**Conclusions:**

The established ‘measure and manage’ approach to patient safety is difficult to apply to care which is geographically dispersed and delivered by practitioners in multiple professions with overlapping scopes of practice, primarily in a fee-for-service model. Collaboration across professions on models that allow practitioners to share information anonymously and help practitioners learn from the reported incidents is needed.

## Background

Spinal manipulation therapy (SMT) is a non-invasive, manual procedure applied to specific body tissues with therapeutic intent. Estimates suggest that 50% of Canadians have undergone SMT, most commonly for back or neck pain [1, 2]. Amongst North American regulated health professionals, SMT is most often delivered by chiropractors, and to a lesser extent, by a specially-trained subset of physiotherapists and osteopathic practitioners [2]. It is also included in the scope of practice of naturopaths (NDs) and physicians (MDs) although, based on our Canadian data, these providers rarely use SMT as their primary treatment modality.

While adverse events associated with SMT have been reported in the literature,^2^ evidence of incidence varies [3, 4]. Moreover, this evidence is often of low quality and has sometimes been the subject of rancorous debate [3, 5–10]. In addition, evidence about SMT safety is often eclipsed by anecdotal beliefs emerging from inter-professional rivalries and sensationalized media coverage of rare, catastrophic events [11]. There is also no consensus on how to define adverse events associated with SMT [12–15]. However, evidence informed documents have been produced to assist the clinician in the understanding of adverse events in cervical spine manipulation [16]. Yet despite SMT’s popularity and the increasingly global focus on patient safety in healthcare, there are currently few formal safety and reporting mechanisms to address adverse events related to SMT [17].

This study represents an early phase of a multi-phased program of research which included the development and assessment of a prospective active surveillance and learning system for practitioners who provide spinal manipulation therapy (SMT). The purpose of this study was to gain insight into the current safety culture around the use of spinal manipulation therapy by regulated health professionals in Canada and to explore perceptions of readiness for implementing formal mechanisms for tracking associated adverse events.

### Thinking about patient safety in relation to SMT

Our starting point for considering patient safety in relation to SMT was James Reason’s [18] widely familiar Swiss cheese model of risk which focuses on weaknesses within systems rather than individual actors as the agents of harm. Derived from high-risk industry, this model, (which is dominant in healthcare), maintains that accidents result from the alignment of latent conditions and active failures, and it has shaped the evolution of patient safety culture within the acute hospital sector for more than 15 years.

The application of Reason’s model to patient safety outside the acute hospital setting presents a number of challenges. For example, the widely dispersed and lower acuity primary care environment differs from the acute hospital setting and has its own, indigenous risks [19–21]. Medical primary care is delivered in geographically dispersed and varied practice settings through an array of administrative structures, communication systems and professional networks. Medical primary care providers also deal with a multitude of undifferentiated presentations and are often required to care for patients in the absence of a diagnosis. Thus, in contrast with acute hospitals, where concern has typically been focused on risks such as medication errors, falls and hospital-acquired infections, risks to patient safety in medical primary care are primarily linked to administrative and communication issues and to long-term medication management [19].

Recognition of risks indigenous to primary care has been reflected in the adaptation or development of dedicated approaches to patient safety, such as the Primary Care Hazard Permeation (PCHP) model [19], and The Manchester Patient Safety Framework for primary care (MaPSaF) [22]. The PCHP is a primary care version of the Swiss cheese model where patient outcomes pass through the latent layers of organizational leadership, management, and situations for safe practice, and the active layers of provider and patient performance [19]. The MaPSaF assesses local safety cultures by drawing on Ron Westrum’s model of organizational engagement [23]. It describes a range of attitudes toward patient safety ranging from the Pathological (“Why are we wasting time on this?”); to the Reactive, (“We take patient safety seriously and do something when we have an incident”); to the Bureaucratic, (“We have systems in place to manage patient safety”); to the Proactive, (“We are always thinking about patient safety issues …”) and, finally, the Generative, (Managing patient safety is an integral part of everything we do).

Non-medical primary care, the usual setting for SMT, diverges even further from the acute hospital context and the high-risk industries that inspired Reason’s approach to patient safety. Unlike hospital-based care, non-medical primary care is unbounded, as SMT is delivered by thousands of independent practitioners across several regulated (and an indeterminate number of unregulated) professions. Each regulated profession has governance structures, requirements for licensure, regulations and standards of care, but these are not subject to inter-professional agreement or structured information sharing. Moreover, many practitioners delivering SMT work in small group or solo practices, some in geographically remote locations. So while practitioners may adhere to the rules and regulations governing their profession, participation in a shared safety culture of any kind poses clear challenges but also an opportunity for interprofessional collaboration.

### The ‘measure and manage’ approach to patient safety

Safety culture in hospitals is dominated by a ‘measure and manage’ approach [24] that requires a clearly defined set of measurable outcomes, effective systems for carrying out measurement, and an environment that treats errors as opportunities for learning rather than as cause for blame. “A key feature of this approach is that knowledge about risk is widely treated as objective, established through scientific classification, and amenable to management exploitation in the pursuit of organisational learning” [24] (p.17220). Put another way, the ‘measure and manage’ approach assumes a positivist stance in which objective knowledge about safety and risk can be used to enable preventive action, iconically, through the use of incident reporting systems. However, as Justin Waring argues, such knowledge is easily confounded, for rather than simply being derived from scientific evidence, “knowledge about safety is constructed and re-constructed through inter-subjective story telling and through interaction with … [healthcare] systems” [23] (p.1723). This scenario is considerably more complex than the phrase ‘measure and manage’ implies especially in a geographically dispersed, inter-professional context characterized by longstanding intra and inter-professional disagreements, anecdotally-based beliefs about safety standards within and between professions, and a long history of negative attitudes toward chiropractic [11]. The case of SMT thus provides an opportunity to re-consider approaches to patient safety in the light of this complexity.

## Methods

The ideas presented here emerged from the qualitative arm of the SafetyNET study, a five year, multi-disciplinary project exploring safety issues related to SMT [25]. As healthcare is provincially regulated in Canada, the focus of the study was on the regulated health professional groups whose scope of practice included SMT in the provinces of Alberta and Ontario. However, because professional leaders often engage with issues through inter-provincial discussion and may hold positions in more than one province over the course of their careers, the geographic scope was broadened to national recruitment in these cases. This was also the case in recruiting naturopathic doctors because this is a small profession in Canada. Ethical approval was received from the Research Ethics Boards at the Universities of Alberta and Toronto.

### Recruitment

Fifty-six semi-structured telephone interviews were conducted with professional leaders and frontline practitioners in chiropractic, physiotherapy, naturopathy and medicine between October 2011 and October 2013. Individuals in leadership roles across all participating professions, Canada-wide, were identified through professional networks and the websites of regulatory colleges, educational institutions and professional associations. This was supplemented by snowball recruiting in cases where the individuals initially identified were no longer in leadership roles or indicated they were unable to address the issues under study.

A list of all frontline chiropractors in the province of Alberta was obtained from the regulatory body. A stratified random sample was then compiled based on sex, number of years in practice and urban vs rural location. Physiotherapists rostered to perform SMT were identified through publicly available sources such as association and regulatory body lists. Naturopathic frontline practitioners were identified by searching publicly available information and practice websites for information about the range of treatments offered. Individuals whose practice websites included reference to manual therapies were invited to participate.

Consent forms and information letters outlining the study were sent to prospective participants by email. If no response was received, this was followed up with up to three phone calls. Signed consent was returned to the qualitative researcher by fax prior to interview. Participants had the opportunity to ask questions about the study at every stage of the recruitment process and immediately prior to their interview.

### Data collection

Data collection took place between October 2011 and October 2013. All interviews were conducted by an independent, experienced qualitative researcher (LR) who is not a clinician and has no vested interests in the provision of SMT. Interviews were digitally audio recorded for verbatim transcription. All personal identifiers were removed from transcripts and all transcripts were verified by the interviewer and corrected where necessary. Interviews lasted between 13 and 60 min with an average length of 45 min.

Interviews with professional leaders explored: perceptions of the main safety issues around SMT; the locus of responsibility for SMT safety; risk and liability management; the current safety culture around SMT; perceptions of SMT safety as practiced by their own and other professions; the potential value of a formal adverse event reporting and learning system; and public perception and media coverage of SMT. In contrast, interviews with frontline practitioners explored: provision of treatment information to patients; risk disclosure and informed consent processes; practitioner experience of adverse events related to SMT; current practices for reporting adverse events and disclosing harm; professional support for practitioners following an adverse event; perceptions of SMT safety in their own and other professions; the potential value of a formal adverse event reporting and learning systems, and suggestions for optimizing SMT safety.

In total, we interviewed 55 practitioners including 23 professional leaders and 32 frontline practitioners. Table 1 provides key demographic characteristics of those who participated in the study.

**Table 1:** Participant demographics

	Professional leaders			Frontline providers		
Profession	No.	Average years in practice	No. male/female	No.	Average years in practice	No. male/female
Chiropractic	8	27.8	5/3	13	12.7	11/2
Physiotherapy	6	12	3/3	7	19.1	4/3
Naturopathy	7	20.3	5/2	9	10.8	3/6
Medicine	2	20.5	2/0	3^a^	37.3	3/0

^a^includes 1 MD/chiropractor

### Analysis

All transcripts were read and annotated independently by three members of the study team. Ongoing discussion throughout the data collection and post collection periods identified anticipated and emergent themes which were used to inform development of the formal coding framework. Formal coding was conducted by the qualitative researcher and was then used to produce a thematically organized, descriptive analysis.

## Results

Among our most striking findings were: a matrix of complex, competing narratives that simultaneously informed and clouded the safety culture around SMT, and multiple barriers participants anticipated to the use of an incident reporting system. These will be discussed in turn beginning with the three distinct themes which illustrate the complexity of competing narratives around SMT safety.

### Narratives of intra-professional disagreement

Intra-professional disagreements were evident within all participating professions but were most striking in the case of chiropractic. Mainstream chiropractic in Canada today is focused on the “assessment, diagnosis, treatment and preventative care of biomechanical disorders originating from the muscular, skeletal and nervous systems” [26]. However, some within the profession continue to espouse an unorthodox view, founded on the notion that the subluxation is an obstruction to health, [27] a notion that is rejected by those who identify as evidence-based chiropractors [28, 29]. Evidence-based chiropractors consider such colleagues to be diminishing safety standards and the profession’s reputation. As a result, many were keen to distance themselves from this group:

*This profession … is discordant … and one of the splinter groups relies heavily on dogma and unscientific information. They have a specific agenda, and for some reason they paint the profession with a variety of unscrupulous practice methodologies, and the media and patients and the public are quick to pick up on this and generalize that across the profession at large and I think that hurts the profession’s image. [Chiropractic prof. leader 01]*

The use of SMT to treat a broad range of conditions and questionable business practices that might lead to excessive or inappropriate use of SMT were seen as clouding the safety picture and creating an impression of danger associated with chiropractic:

*I think making outlandish claims for a simple spinal manipulation is something that I have to fight quite often in the office. Somebody says, “Hey, cervical dystonia, sure, all you need is fifty treatments from a chiropractor.” Show me the research, you know? [Chiropractor 20]*

### Narratives of inter-professional disagreement

Competing claims about SMT safety were similarly evident across all participating groups but were most pronounced in the relationship between chiropractic, which delivers the majority of SMT, and physiotherapy, which is the second most frequent provider of the treatment [2]. Here, seemingly intractable beliefs about each other’s education and clinical practice further clouded the debate about the actual risks of SMT.

While a handful of participants said that they lacked sufficient knowledge to comment on safety standards outside their own profession, many argued assertively that the level of safety they were able to achieve exceeded that in the other profession even while admitting that their knowledge of the other professions was anecdotal and limited. Chiropractic participants typically saw themselves as the SMT specialists based on their education and the consistent volume of treatment delivered. This was contrasted with SMT-certified physiotherapists whom they characterized, often using emotive language, as inadequately trained and amateur:

*I think of how much of a skill and art it is actually to do a chiropractic adjustment. It’s just like a wood carver. It’s either you’re swinging an axe to cut a tree down or you’re having a fine chisel and I think that’s the difference. It takes thousands of adjustments and thousands of hours of just doing it and plus the learning behind it is significant. [Chiropractor 12]*

*Do you think that a master mechanic could fix your car better than the back yard garage mechanic? I mean, that’s a no-brainer. [Chiropractor 17]*

Moreover, the erroneous belief that physiotherapists learned to perform SMT over the course of a weekend was widespread amongst chiropractic participants. While SMT training for physiotherapists is partly delivered on weekends, since this is post-graduate training for working professionals, it is not a single weekend course [30].

*When we [chiropractors] do manipulation training it’s a minimum of an hour a day every day for four years. I know guys [physiotherapists] that have taken a weekend course and done manipulation with somebody in the office the next day. [Chiropractor 20]*

On the other side, many physiotherapists argued that the basis of SMT safety was not length of training or volume of treatment delivered, but patient selection and appropriateness of application. Hence, they maintained that their more varied approach to treatment was superior to that of chiropractors who, they believed, used the technique indiscriminately and were simply displaying territoriality in identifying themselves as the SMT specialists. References to SMT being just one of the tools in the physiotherapist’s toolbox were commonly offered in support of this view. Many also cited the practice of screening patients for stroke risk using a pre-manipulative test of vertebrobasilar insufficiency [31] as proof of the superior safety standards in physiotherapy. Interestingly, this argument was even made by some who acknowledged that this kind of pre-manipulative testing was a weak indicator of safety. The representation of chiropractors as imprudent practitioners with only one thing to offer was a striking feature in these descriptions. Again, the language is remarkably emotive:

*Compared to chiropractors we are definitely safer because we at least try to screen people in and out and we don’t go with guns blazing. And it is one of the tools that we have to offer so it’s not our only tool. [Physiotherapist 05]*

*You use neck manipulation all the time and somehow you say that you’re safer than someone who uses it more judiciously as part of a well-rounded practice where they incorporate self-management and education of the patient and empowerment of health and exercise. I would say that that is a much healthier way of practicing than cracking necks all day long all the time, week-in and week out for the same patient. [Physiotherapist 11]*

While these beliefs (many of which were attributed to sources such as patient anecdotes, general hearsay and videos posted on the internet) tell us little about clinical safety, they reveal a great deal about the power of discourse in constituting the current safety culture around SMT:

*Somebody’s been seen for a minute, manipulated in the neck and off they go, no word to a lie. They’ve been in somebody’s office for four or five minutes and they’re manipulated here, there and there to the neck and off they go. [Physiotherapist 07]*

*Any time there’s ever been talk about manipulation and strokes it always came from other disciplines doing work on the spine. Everything that I’ve heard about that has been documented or brought up in regard to that, it’s always been it happened through a medical doctor doing it, it happened through a physio doing it. [Chiropractor 12]*

### Narratives conflating business competitiveness and clinical risk

To varying degrees, chiropractors, physiotherapists and naturopathic doctors have overlapping scopes of practice and compete for discretionary healthcare spending in a rapidly expanding and competitive CAM market. This gives rise to “protection of scope, protection of client, [and] protection of customer” [Naturopathic prof. leader 04]. Unsurprisingly, competition for business and discussion of risk were often conflated adding further complexity to the SMT safety landscape:

*It’s a competitive marketplace out there so if I can scare you off of the chiropractor you’re more likely to spend your dollars in my office. I think physiotherapists are trying to exclude chiropractic from involvement in many things and one of the tools that they have to do that is fear of spinal manipulation of the neck. [Chiropractor23]*

One place this dynamic was especially evident was in the relationship between chiropractors and naturopathic doctors. While the origins of modern naturopathic medicine in Canada are closely linked with chiropractic [32, 33], the profession has evolved in other directions and only a handful of naturopathic participants in our study used SMT as a primary treatment modality. That said, SMT is still a required competency for naturopathic licensure and remains within the naturopathic scope of practice.

Although many NDs viewed chiropractors as the SMT specialists, they resented being portrayed as unsafe because they believed such portrayals were due to protectionism:

*There is a bit of a turf war, “Well, those are my patients. You shouldn’t be treating those patients with that type of therapy because that’s what I do and that’s what I’ve been specialized and trained to do.” I understand where a person could be coming from but to say that what you do is the only way it should be done, that’s wrong because if there are other licensed healthcare professions that allow that, there must be a reason. Otherwise, it wouldn’t be part of their scope of practice. It never would have passed legislature. It never would have been licensed. [Naturopath 08]*

*It’s very easy to say it’s for the public and for safety especially when you don’t understand the strength of our program and then say that it’s not enough. If it wasn’t enough we wouldn’t be using it. I know there’s a movement to take the modality away from us. They will say that it’s because the training is inadequate. [Naturopath prof. leader 02]*

This conflation of competitiveness and safety talk was especially irksome to naturopaths because they believed chiropractors were increasingly moving into the naturopathic scope of practice, for example, through the use of acupuncture and nutritional counseling. Some participants expressed concern because they believed that chiropractors had minimal training in these other modalities:

*You’ve got chiropractors learning acupuncture and when they get out into private practice, they’re doing acupuncture with less training than what the naturopaths have but they’re still able to do it. So what’s the big kerfuffle about naturopaths doing manipulation? [Naturopath 03]*

*I know here in Alberta, chiropractic medicine was de-listed about four years ago. They’ve got to make up that income loss and so a lot of them are getting into nutrition and prescribing supplements. They’re almost wanting to practice like naturopaths. If you want to practice like a naturopath, go back and become a naturopath. [Naturopath 01]*

### Responses to a proposed incident reporting system for SMT

While the prospect of having better data to support better practice was broadly welcomed by participants, the perceived barriers to implementing an incident reporting system for SMT were legion. Some of these were practical barriers linked to the unbounded and multi-professional nature of SMT delivery. Namely, how do you implement and administer a tracking system involving thousands of geographically-dispersed practitioners across multiple professions? Other perceived barriers reflected concerns about the potential for recriminatory use of information given the inter- and intra-professional differences that currently exist. But beyond these logistical and socio-cultural concerns, there were fundamental questions about the workability of an incident reporting system for adverse events associated with SMT.

To begin, simply defining reportable adverse events associated with SMT was considered a challenge. Many of the commonly experienced after-effects of spinal manipulation [34], such as additional stiffness and soreness, were considered by participants to be minor, transient and expected. As such, there was broad agreement that the impracticality of tracking such events would far outweigh the benefits of having the information:

*So, depending on what your definition is, 50% of my patients could have an adverse reaction after the first consultation. Because it’s often that people have more soreness when you do an assessment and attempt to provoke different structures to help figure out what’s going on. [Physiotherapy prof. leader 09]*

*You’re using a physical therapy on joints and tissues that are already sore so is soreness after a treatment for 24 or 48 hours an adverse outcome, or is it part of what you would expect in terms of applying that energy to an injured, already sore tissue? [Chiropractic prof. leader 05]*

Determining an appropriate interval for the duration of adverse symptoms was considered equally problematic. Estimates of what might distinguish a transient effect from one of enduring significance were both highly impressionistic and highly variable:

*Well, certainly anything that has a long-term or permanent effect would have to be reportable. What about something that bothers you for a month but then gets better? Is that worth knowing about? You don’t see that very often. I think anything that persists longer than a week maybe. I’m just kind of pulling that out of the air. [Osteopathic prof. leader 11]*

*Twenty-four to 48 hours is too much, yet three hours is fine. It’s hard to say, right? [Physiotherapy prof. leader 08]*

At the other end of the spectrum, catastrophic events such as strokes were so rare that it was felt there would be similarly limited value in tracking them. Interestingly, the question of what, if anything, might constitute a usefully trackable mid-spectrum event proved elusive.

Another perceived barrier to implementing an incident reporting system for SMT was that practitioners might not even know when an adverse event had taken place. Patients receiving SMT are ambulatory and their complaints are generally of lower acuity than conditions requiring hospital-based care. Thus, unintended after-effects of treatment might not be evident for hours or even several days after treatment [35] and practitioners would only become aware of such events if patients returned or otherwise alerted them to the situation. Furthermore, because CAM is something that people seek out at their own discretion and often pay for out of pocket, it is common for patients to simply discontinue treatment if they are dissatisfied with the experience, start to feel better, or are deterred by cost. This makes it difficult, if not impossible, to interpret why they are lost to follow-up:

*Sometimes you don’t know because a patient will discontinue care and so how do you know why they discontinued? You may find out six months later when you get a letter from a lawyer that there was a harm perceived by the patient from care, or the patient may have just decided to pursue another type of therapy so there was no harm, they just didn’t think it was helping. I don’t know how you would get an accurate system because the reporting is going to be skewed no matter what. [Chiropractic prof. leader 05]*

Similarly, determining the frequency of events was thought to require prohibitively labour-intensive follow-up:

*Do you go out of your way to follow up with people two, three weeks later? Do you call them and say, “By the way I’m just following up on our treatment on this day. Can you tell me how you’re feeling these days? [Physiotherapist 15]*

Finally, some participants pointed out that, in order to be useful, an incident reporting system for SMT would need to capture sufficient clinical context, such as the broader case history and specific treatment techniques used and that, in the absence of such detail, the information collected would be inactionable.

*If we call it spinal manipulative therapy that makes an inherent assumption that it’s one type of thing that’s happening. There are many different ways to address the different segments of the spine. [Chiropractor 22]*

*So much in the literature is documented with adverse effects but where it falls down is the type of manipulation that was used. Was it rotary, was it translatory, was it longitudinal? So I think a database would be helpful, but making sure that we’re documenting the vectors that were used. [Physiotherapist 07]*

## Discussion

The findings of this study illustrate some of the challenges associated with applying the dominant model of patient safety to the realm of non-medical primary care exemplified by the case of SMT. Specifically, they lead us to consider how best to address issues of patient safety in a context where the actual (as opposed to putative) risks of a practice are a matter of ongoing debate, and where competing narratives of risk and safety further cloud the safety picture. These findings also identify challenges with the implementation of incident reporting systems in the context of care which is geographically dispersed, institutionally unbounded and delivered by multiple professions with overlapping scopes of practice. In addition, our findings suggest that models such as Westrum’s [23], which address the cultural context of safety, may be unhelpful when they read the failure to embrace a given idea of safety culture as a sign of institutional immaturity rather than of unacknowledged complexity.

Other commentators have similarly identified challenges with applying a ‘measure and manage’ approach to patient safety outside the acute hospital setting [36, 37]. Challenges in defining errors in primary care, under-reporting of safety incidents, a lack of recognition of reportable events and the divergent logics governing risk management and medical practice have all been noted in relation to both medical and allied primary care [38–42]. A call for new approaches to patient safety has also emerged from the field of homecare [43] and some have argued that the safety gains made using a ‘measure and manage’ approach have been modest even within the acute hospital setting [44, 45].

Experience emerging from a number of European chiropractic incident reporting and learning initiatives both aligns with our findings and describes efforts to move beyond the familiar impediments of under-reporting, non-recognition of reportable events, fear of recrimination and time burden to practitioners [46–50]. Currently underway, the Canadian SafetyNET initiative, incorporates multi-phased strategies involving stakeholders from a range of professions to identify challenges and opportunities in promoting patient safety, assessing safety culture and developing and implementing specific incident reporting and education protocols [25]. This initiative can be used to model interprofessional collaboration amongst SMT regulated professions to work together to address the identified challenges herein and develop a common approach to patient safety that transcends the unique profession’s philosophy.

Finally, the findings of this study confirm the value of more socially-grounded and context specific approaches to patient safety [51–54] and encourage us to ask a different set of questions posed from an interprofessional perspective. For example, “How can professions collectively address patient safety in dispersed, multi-professional care delivery? How can the safety talk be separated from business competitiveness in fee-for-service environments? How should one choose which risks to focus on and how will they know that they are focusing on the right things? And perhaps, most importantly, as the ways in which healthcare delivery evolve, they encourage us to ask, “What alternatives can be imagined to a measure and manage approach to patient safety?”

## Conclusion

The established approaches to patient safety derived from high-risk industry and commonly used in acute hospital settings are difficult to apply to non-medical primary care. Collaboration across professions on models that allow practitioners to share information anonymously and help practitioners learn from the reported incidents is needed.

## Abbreviations

MaPSaF, the Manchester patient safety framework for primary care; NDs, naturopaths; PCHP, primary care hazard permeation; MDs, physicians; SMT, spinal manipulation therapy
